# Stress Tolerance and Ecophysiological Ability of an Invader and a Native Species in a Seasonally Dry Tropical Forest

**DOI:** 10.1371/journal.pone.0105514

**Published:** 2014-08-19

**Authors:** Marciel Teixeira Oliveira, Virginia Matzek, Camila Dias Medeiros, Rebeca Rivas, Hiram Marinho Falcão, Mauro Guida Santos

**Affiliations:** 1 Department of Botany, Federal University of Pernambuco, Recife, Brazil; 2 Department of Environmental Studies and Sciences, Santa Clara University, Santa Clara, California, United States of America; University of Saskatchewan, Canada

## Abstract

Ecophysiological traits of *Prosopis juliflora* (Sw.) DC. and a phylogenetically and ecologically similar native species, *Anadenanthera colubrina* (Vell.) Brenan, were studied to understand the invasive species’ success in caatinga, a seasonally dry tropical forest ecosystem of the Brazilian Northeast. To determine if the invader exhibited a superior resource-capture or a resource-conservative strategy, we measured biophysical and biochemical parameters in both species during dry and wet months over the course of two years. The results show that *P. juliflora* benefits from a flexible strategy in which it frequently outperforms the native species in resource capture traits under favorable conditions (e.g., photosynthesis), while also showing better stress tolerance (e.g., antioxidant activity) and water-use efficiency in unfavorable conditions. In addition, across both seasons the invasive has the advantage over the native with higher chlorophyll/carotenoids and chlorophyll a/b ratios, percent N, and leaf protein. We conclude that *Prosopis juliflora* utilizes light, water and nutrients more efficiently than *Anadenanthera colubrina*, and suffers lower intensity oxidative stress in environments with reduced water availability and high light radiation.

## Introduction

The caatinga forests of northeastern Brazil harbor a unique and specialized flora, with 34 percent endemic species among the woody plants and succulents, that require more conservation attention [1]. Like other seasonally dry tropical forests, caatinga exhibits an unusual set of ecological conditions, with a brief, warm rainy season ideal for plant productivity, and a harsh dry season with virtually no rainfall. Precipitation is extremely irregular, and the high interannual variability results in occasional prolonged droughts [2]. Caatinga canopy vegetation typically consists of thorny scrub or small trees, with drought-deciduous leaves and roots adapted to efficiently capture water in shallow soil layers [3], while the understory is characterized by life forms that can escape drought phenologically, tolerate desiccation, or store water in their tissues [4].

Invasion biologists have long been interested in whether there is a complex of plant traits that differs systematically between invasive and non-invasive species [5], [6]. Initial attention was focused on the classically “weedy” habits like fast growth, high photosynthetic rates, and high specific leaf area, all good strategies for exploiting high resource availability [7], [8]. However, evidence has been accumulating that some exotics invade low-resource habitats by exhibiting the traits associated with persistence and survival under harsh environmental conditions, such as resource-use efficiency and resistance to herbivory [9]–[11]. We wondered, does invasion by woody plants in this seasonally dry forest occur because invaders are more able to take advantage of high resource availability in the rainy season, because they are more able to tolerate low resource availability in the dry season, or both?

We focused on *Prosopis juliflora,* a particularly widespread invader of tropical dry forests and savannahs around the globe. *P. juliflora* (common names: mesquite, algaroba), is native to Central America, northern South America and the Caribbean Islands [12] and was introduced in Brazil as a supplementary food for grazing animals. *P. juliflora* has been classified as an aggressive alien and now occupies millions of hectares in Africa, Australia, and Asia [13]. *Prosopis* invasion has a profound impact on caatinga forest biodiversity, as it dramatically decreases the richness of native trees and shrubs [14] and compromises the ability of native vegetation to regenerate [15]. To simplify our comparisons with the native caatinga flora, we paired our observations of *P. juliflora* with a phylogenetically and ecologically similar tree species that was, prior to environmental degradation and invasion, a canopy dominant: *Anadenanthera colubrina* [16]. Both are small, leguminous trees that reproduce primarily by seed [12], [17].

To understand if the success of *Prosopis* is due to better drought-tolerance during the dry season or superior capture capacity during the rainy season, we compared its performance to that of the native *Anadenanthera* in water relations, gas exchange, biochemical components of leaf photosynthesis, and antioxidant metabolism over two years of wet and dry seasons. In arid and semiarid environments, one of the first physiological mechanisms that allows plants to tolerate or escape drought is stomatal control, which reduces water loss through transpiration [18], [19]. Another important component is the primary metabolism of the plant, which figures in adaptation to reduced water potential and the capture of reactive oxygen species via enzymes belonging to the antioxidant protection apparatus [20]. Additionally, the chloroplastidic pigments can change concentration to avoid irreversible photochemical damage and an increase in reactive oxygen species [18], [19]. We hypothesized that superior stress tolerance, or better maintenance of function in the face of worsening conditions, would manifest itself as: 1) smaller dry-season increases in indicators of stress, free radical synthesis, and membrane degradation (proline, peroxide, malondialdehyde); 2) larger dry-season increases in antioxidant enzymes; 3) larger dry-season increases in instantaneous water-use efficiency; 4) smaller dry-season decreases in shoot water potential, 5) smaller dry-season increases in leaf accumulation of excess metabolites as amino acids and soluble carbohydrates; and 6) smaller dry-season decreases in pigment concentrations and in the chlorophyll a/b ratio and chlorophyll/carotenoid ratio. On the other hand, we hypothesized that superior resource capture ability, or better ability to ramp up function to exploit ameliorating conditions, would manifest itself as: 1) larger rainy-season increases in photosynthetic rate and conductance; and 2) larger rainy-season increases in nutrient concentration, starch, and total protein.

## Materials and Methods

**Figure 1:**
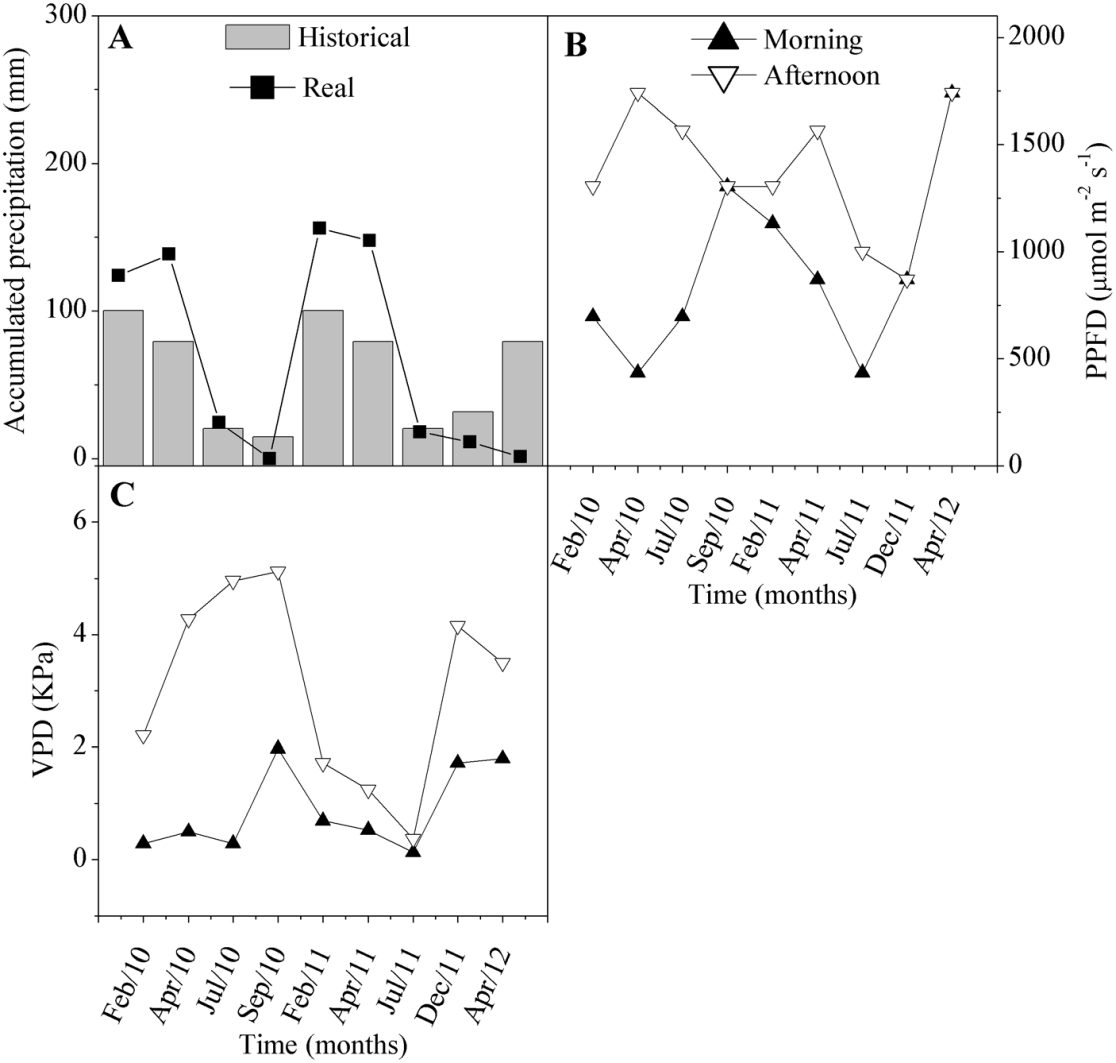
Environmental variables in Serra Talhada (Pernambuco, Brazil) over two years of measurements: (A) monthly total precipitation, (B) photosynthetic photon flux density (PPFD), (C) vapor pressure deficit (VPD). Historical precipitation data represent the years 1999–2011 (source: Agritempo, http://www.agritempo.gov.br).

We sampled both tree species at an altitude of 429 m in the Serra Talhada region in the Brazilian state of Pernambuco (7°54′35″S, 38°17′59″W). The climate type is BSh, according to the Köppen-Geiger classification [21], with an average annual rainfall of approximately 750 mm, and the soil is classified as silt loam. To capture the range of responses to different rainfall conditions in this highly dynamic ecosystem, we sampled five adult plants of each species on nine different dates between February 2010 and April 2012. Measurements were made on different individuals at each sampling date. We later grouped these data into “rainy” months (February 2010, April 2010, February 2011, April 2011) and “dry” months (July 2010, September 2010, July 2011, December 2011, April 2012) based on the cumulative amount of precipitation during each month (Fig. 1a). At the September 2010 sampling date, the native species had already lost its leaves, and consequently, only invasive trees were sampled at that time.

### Biophysical Parameters

The xylem water tension was measured with a Scholander pressure chamber (Soilmoisture Equipment Corp., Santa Barbara, CA, USA) on a branch with a fully expanded, but not senescent, leaf from the middle shoot of the plant. Measurements were performed in the morning and in the afternoon, at 0700 h and 1400 h, and were assumed to measure the leaf water potential (Ψ_l_). Gas exchange was measured using a portable infrared gas analyzer (IRGA, ADC, model LCi-pro; Hoddesdon, UK). Leaf gas exchange was measured from 0800 h to 0900 h (morning) and 1400 h to 1500 h (afternoon) at a constant photosynthetic photon flux density (PPFD) that had been matched to the daýs ambient conditions on a mature, but not senescent, fully expanded leaf in the middle of the plant, using the leaf cuvette of the IRGA. Water-use efficiency (WUE) was calculated by dividing CO_2_ assimilation (*A*) by transpiration (*E*). Fig. 1 shows the ambient average photosynthetic photon flux density (PPFD), cumulative and historical monthly precipitation, and vapor pressure deficit (VPD) for each measurement date.

### Biochemical Parameters

Immediately after gas exchange measurements, leaf samples sufficient to yield ∼3 g fresh weight were collected and frozen in liquid nitrogen and stored at −80°C in humidity-proof containers until analysis. The content of chlorophyll *a*, *b* and carotenoids were analyzed by macerating 80 mg of leaf tissue in 2 ml of acetone (80%) with CaCO_3_ to prevent chlorophyllase activity. After maceration, the samples were filtered and read on a spectrophotometer at absorbances of 470.0, 646.8, and 663.2 nm. Additionally, a nonspecific absorbance at 710 nm was recorded to correct for color, turbidity, and contaminating compounds, as Chl *a*, *b* and carotenoids do not absorb this wavelength. Final pigment concentrations were calculated as described by Lichtenthaler and Buschmann [22]. For extraction and measurement of free amino acids (AA), soluble carbohydrates (SC) and starch, 10 mg of leaf tissue was used to prepare the ethanol extract. Soluble carbohydrates were measured according to Dubois et al. [23], using D(+)-glucose as a standard. Free amino acid analyses were performed according to Moore and Stein [24], using a 1 mM solution of glycine, glutamic acid, phenylalanine, and arginine as a standard. The insoluble fraction from the extraction of carbohydrates was used to determine starch content; the pellet was hydrolyzed for one hour with 10 units of amyloglucosidase, and the resulting sugars analyzed a second time [23], using D(+)-glucose as a standard. To determine the leaf protein (TP) content, we extracted 50 mg of leaf tissue in a buffer consisting of 10 percent (v/v) glycerol, 0.1 percent (w/v) bovine serum albumin, 0.1 percent (v/v) Triton X-100, 50 mM HEPES/KOH (pH 7.4), 5 mM MgCl_2_, 1 mM EDTA, 1 mM EGTA and 5 mM dithiothreitol. Leaf protein content was estimated according to the method of Bradford [25], using bovine serum albumin as a standard. To measure nitrogen, phosphorus, and potassium, leaves were dried at 80°C until constant weight, and a pulverized 250-mg sample was then subjected to extraction with sulfuric acid and the nutrient concentration measured colorimetrically [26].

Total superoxide dismutase (SOD, EC 1.15.1.1) was extracted from 100 mg of leaf tissue in a buffer consisting of 100 mM K-phosphate, 0.1 mM EDTA, 5 mM dithiothreitol, 10 mM 2-Mercaptoethanol, 0.1 percent (v/v) Triton X-100, and 30 percent polyvinylpyrrolidone. Activity was determined by measuring inhibition of the photochemical reduction of Nitro-blue tetrazolium at 560 nm [27]. The enzymes catalase, ascorbate peroxidase, peroxidase, and polyphenol oxidase were extracted from 50 mg of leaf tissue in a buffer consisting 100 mM K-phosphate, 2 mM EDTA, 20 mM ascorbic acid, and 30 percent polyvinylpyrrolidone. Catalase (CAT, EC 1.11.1.6) activity was estimated by measuring the decomposition rate of hydrogen peroxide at 240 nm using an extinction coefficient of 36 mM/cm [28]. The activity of L-ascorbate peroxidase (APX, EC 1.11.1.11) was measured by reducing hydrogen peroxide to water with ascorbate as a reducing agent and by monitoring the decrease in absorbance at 290 nm; enzyme activity was then calculated using the extinction coefficient of 2.8 mM/cm [29]. Peroxidase (POD, EC 1.11.1.7) and polyphenoloxidase (PPO, EC 1.14.18.1) were measured at 420 nm absorbance as the amount of purpurogallin formed; this measure is expressed in units of activity, in which one unit is defined as the change in one unit of absorbance per second [30].

Proline content was measured with the acid-ninhydrin method using 20 mg of leaf tissue [31]. To assess cellular damage, we measured the accumulation of malondialdehyde (MDA) and hydrogen peroxide (H_2_O_2_) using 100 mg of tissue. MDA content was assessed with the thiobarbituric acid (TBA) test, which measures MDA as a final product of lipid peroxidation. The amount of MDA-TBA complex (red pigment) was calculated using an extinction coefficient of 155 mM/cm [32]. Hydrogen peroxide (H_2_O_2_) accumulation was measured spectrophotometrically after reacting with KI [33].

### Statistical Analysis

Data were subjected to factorial ANOVA, and the means were compared using the Student Newman-Keuls test with the significance level set at 5 percent. ANOVA tests of the variables of gas exchange (stomatal conductance, CO_2_ assimilation, transpiration, water use efficiency and water potential) included species (native and invasive), season (dry and rainy) and day time (morning and afternoon) as factors. For all other variables, which were measured in pulverized leaf tissue, species (native and invasive) and season (dry and rainy) were the only factors in the ANOVA. To reduce the experiment-wide probability of Type I error for multiple comparisons resulting from the simultaneous analysis of 25 traits, we used the procedure of Benjamini and Hochberg [34] with an α of 0.05. The data were analyzed using Statistica 8.0 (StatSoft. Inc., Tulsa, OK 74104, USA).

### Ethics Statement

Fieldwork and sample collection were authorized to take place at the IPA Experimental Station in Serra Talhada, Brazil (7°54′35″S, 38°17′59″W), by the Brazilian Environmental Ministry (MMA), under permit number 27017-1. Fieldwork did not involve endangered or protected species.

## Results

Environmental conditions were different between rainy and dry months during the study period (Fig. 1). The total monthly precipitation varied from an average of 141 mm in the rainy months to only 11 mm in the dry months. The photosynthetic photon flux density (PPFD) averaged 783 µmol/m^2^/s^1^ (morning) and 1479 µmol/m^2^/s^1^ (afternoon) in the rainy months and 1000 and 1300 µmol/m^2^/s^1^ in the dry months. The soil moisture went from 8 percent (v/v) during the rainy season and 2 percent in the dry. The dry-season vapor pressure deficit (VPD) averaged 1.17 kPa (morning) and 3.62 kPa (afternoon), while the comparable rainy season figures were 0.50 and 2.35 kPa. This seasonal variation induced changes in the important parameters of gas exchange and photosynthetic metabolism, both biochemical and enzymatic. The attributes that were most responsive to the seasonal variations were gas exchange (g_s_, *A*, *E*, WUE), water potential, and the osmoprotectant proline (Table 1).

**Table 1:** Statistical summary of effects of season, time of day, and species origin (invasive/native) for 25 functional traits.

	Effect						
Attribute	Species	Season	Time	Species vs Season	Species vs Time	Season vs Time	Species vs Season vs Time
g_s_	3.74	**94.28** ** §	**4.02** *	1.93	0.05	**5.27** *	0.32
Ψ	**69.34** ** §	**337.90** ** §	**39.79** ** §	**40.65** ** §	0.01	0.89	0.12
*A*	**38.41** ** §	**200.77** ** §	1.32	**6.44** * §	0.01	2.32	0.74
WUEi	**9.44** * §	**85.98** ** §	**15.82** ** §	3.19	001	0.05	0.05
*E*	**6.04** * §	**451.18** ** §	**65.54** ** §	0.43	015	**54.85** ** §	0.39
SC	**446.40** ** §	**10.10** * §	–	**40.90** ** §	–	–	–
TP	**54.50** ** §	**4.81** *	–	0.64	–	–	–
AA	**53.23** ** §	**4.81** *	–	3.31	–	–	–
Starch	0.94	**31.76** ** §	–	0.10	–	–	–
Chl *a*	1.07	1.11	–	3.37	–	–	–
Chl *b*	**12.00** * §	2.80	–	1.47	–	–	–
Car	**253.64** ** §	0.85	–	2.04	–	–	–
Chl *a*/*b*	**105.73** ** §	0.03	–	2.77	–	–	–
Chl/Car	**253.20** ** §	**16.40** ** §	–	0.01	–	–	–
SOD	**675.49** ** §	2.98	–	2.93	–	–	–
POD	**102.47** ** §	1.79	–	0.97	–	–	–
CAT	**4.67** * §	**4.92** *	–	0.01	–	–	–
APX	**24.43** ** §	**15.12** ** §	–	**6.60** * §	–	–	–
PPO	**58.39** ** §	1.52	–	0.01	–	–	–
MDA	0.51	**21.61** ** §	–	**9.11** * §	–	–	–
H_2_O_2_	**10.88** ** §	**5.67** * §	–	**7.86** * §	–	–	–
Proline	**32.05** ** §	**87.27** ** §	–	**35.12** * §	–	–	–
N	**5.02** * §	1.96	–	0.46	–	–	–
P	1.14	0.03	–	**6.15** *	–	–	–
K	**37.76** ** §	0.03	–	**17.18** ** §	–	–	–

*Boldface denotes significant results.*

* *p<0.05,*

** *p<0.001;*

§ *denotes p-values significant at α = 0.05 when corrected for 25 comparisons according to the Benjamini-Hochberg procedure.*

### Biophysical Parameters

**Figure 2:**
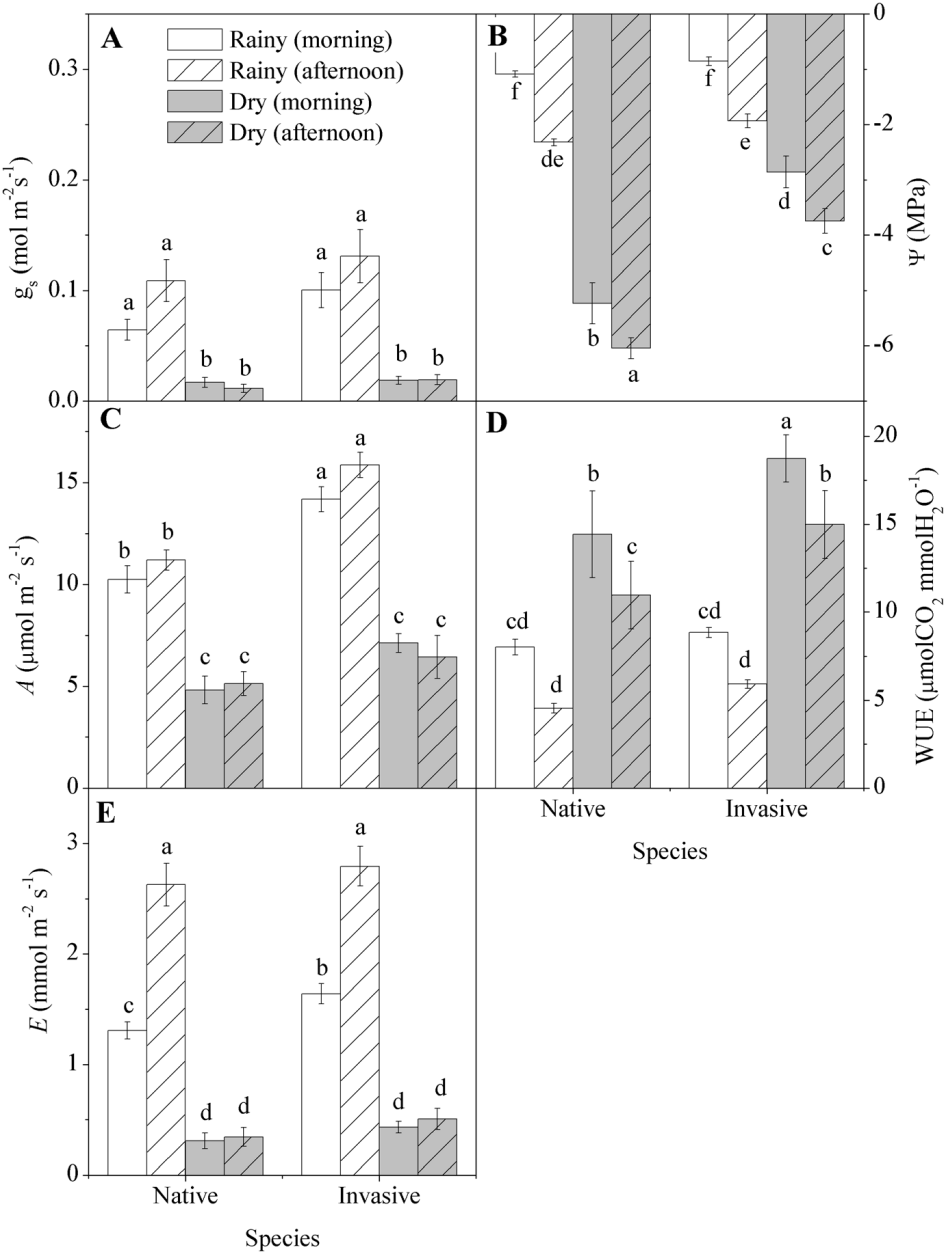
Changes in gas exchange in native (*Anadenanthera colubrina)* and invasive (*Prosopis juliflora*) species in a tropical dry forest in Brazil across seasons: (A) stomatal conductance (g_s_), (B) water potential (Ψ_l_), (C) CO_2_ assimilation (*A*), (D) water use efficiency (WUE = *A*/*E*), (E) transpiration (*E*). Values represent the average of replicates (n = 20±SE; invasive dry season n = 25±SE). Different letters denote statistical differences by Newman-Keul test with significance level of 5 percent between variables.

Gas exchange parameters (Fig. 2) were strongly affected by seasonality and by the time of day of observation. Stomatal conductance did not differ between the native and the invasive species, but both decreased by 83 percent in the dry season (Fig. 2A). In the dry season, the native species had water potentials 60 percent lower than the invasive, and again both species decreased significantly from morning to afternoon (Fig. 2B). The rate of net photosynthesis in the wet season was 38 percent higher in invasive compared to native, but did not differ between species in the dry season, though it declined compared to the rainy period (Fig. 2C). The WUE did not differ between species in the rainy season, but during the dry season the invasive species had values 29 percent and 36 percent higher in the morning and in the afternoon, respectively, compared to the native species (Fig. 2D). In the rainy season, transpiration increased in the afternoon, while in the dry season, transpiration was much 76 percent lower overall, but did not differ between morning and afternoon (Fig. 2E).

### Biochemical Parameters

**Figure 3:**
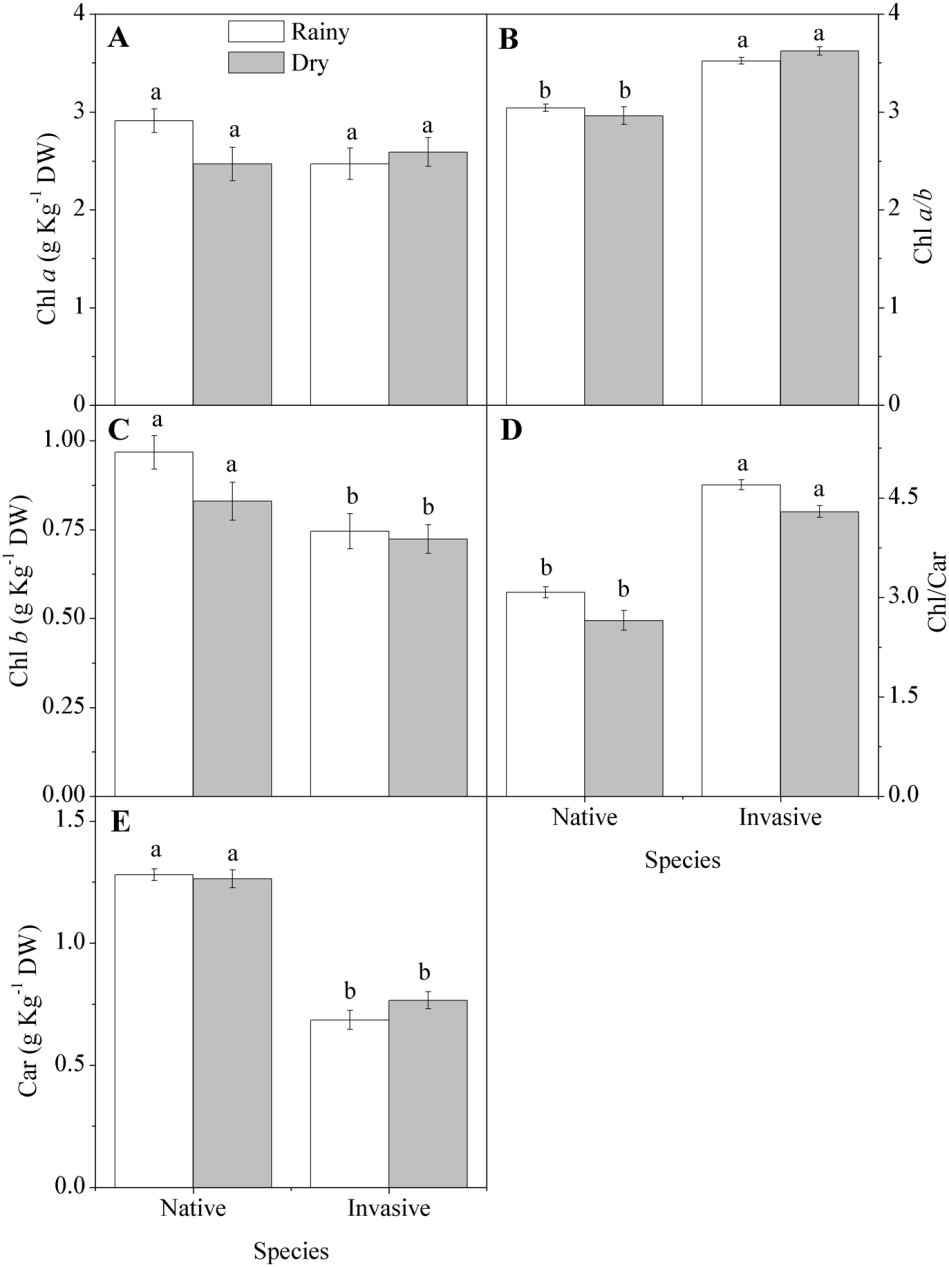
Concentration of leaf pigments in native (*Anadenanthera colubrina)* and invasive (*Prosopis juliflora*) species in a tropical dry forest in Brazil across seasons: (A) chlorophyll *a* (Chl *a*); (B) chlorophyll *a*/*b* (Chl *a*/*b*); (C) chlorophyll *b* (Chl *b*); (D) chlorophyll/carotenoids (Chl/Car); (E) carotenoids (Car). Values represent the average of replicates (n = 20±SE; invasive dry season n = 25±SE). Different letters denote statistical differences by Newman-Keul test with significance level of 5 percent between variables.

Variations between rainy and dry seasons did not promote change in chloroplastidic pigments (Fig. 3). However, pigment concentrations were markedly different between the invasive and native species, with the native species exhibiting 66 percent and 15 percent higher carotenoid and chlorophyll *b* concentrations in both seasons (Fig. 3C and E). Chlorophyll *a* concentrations did not differ between the invasive and the native species (Fig. 3A), but the ratios of chlorophyll *a*/*b* and chlorophyll/carotenoid were 16 percent and 32 percent higher in the invasive species (Fig. 3B and D).

**Figure 4:**
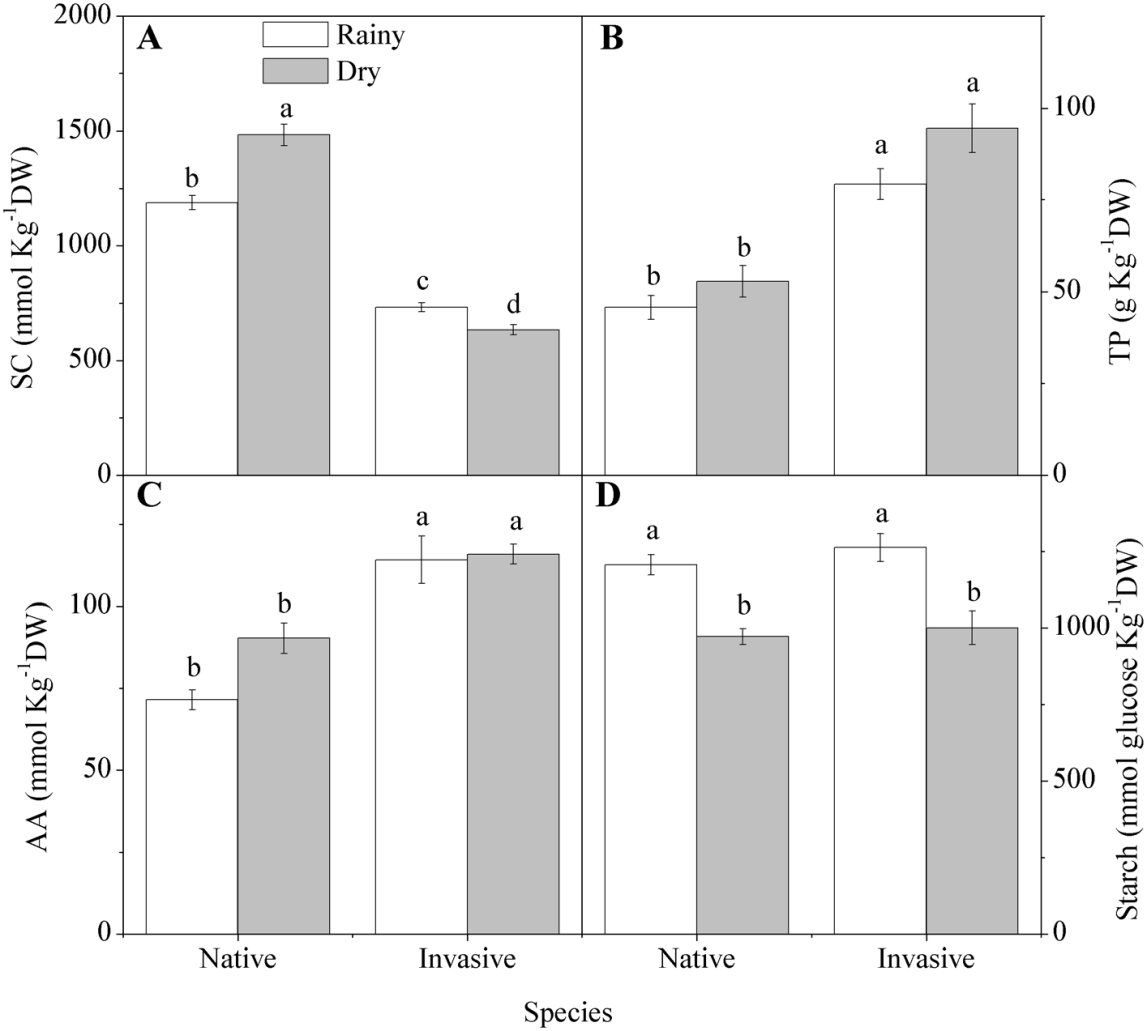
Leaf biochemistry in native (*Anadenanthera colubrina)* and invasive (*Prosopis juliflora*) species in a tropical dry forest in Brazil across seasons: (A) soluble carbohydrates (SC); (B) total proteins (TP), (C) amino acids (AA); (D) starch. Values represent the average of replicates (n = 20±SE; invasive dry season n = 25±SE). Different letters denote statistical differences by Newman-Keul test with significance level of 5 percent between variables.

Soluble carbohydrates showed a significant interaction between species and season, as the carbohydrate concentration increased 24 percent in the native species and decreased 13 percent in the invader from rainy season to dry, although the native species was always higher in carbohydrates than the invader, regardless of the season (Fig. 4A). The concentration of amino acids and proteins did not differ between seasons; however the invasive species had concentrations of 75 percent and 27 percent higher protein and amino acids than the native species (Fig. 4B and C). Starch content did not differ by species but decreased 20 percent in the dry season in both species (Fig. 4D).

**Figure 5:**
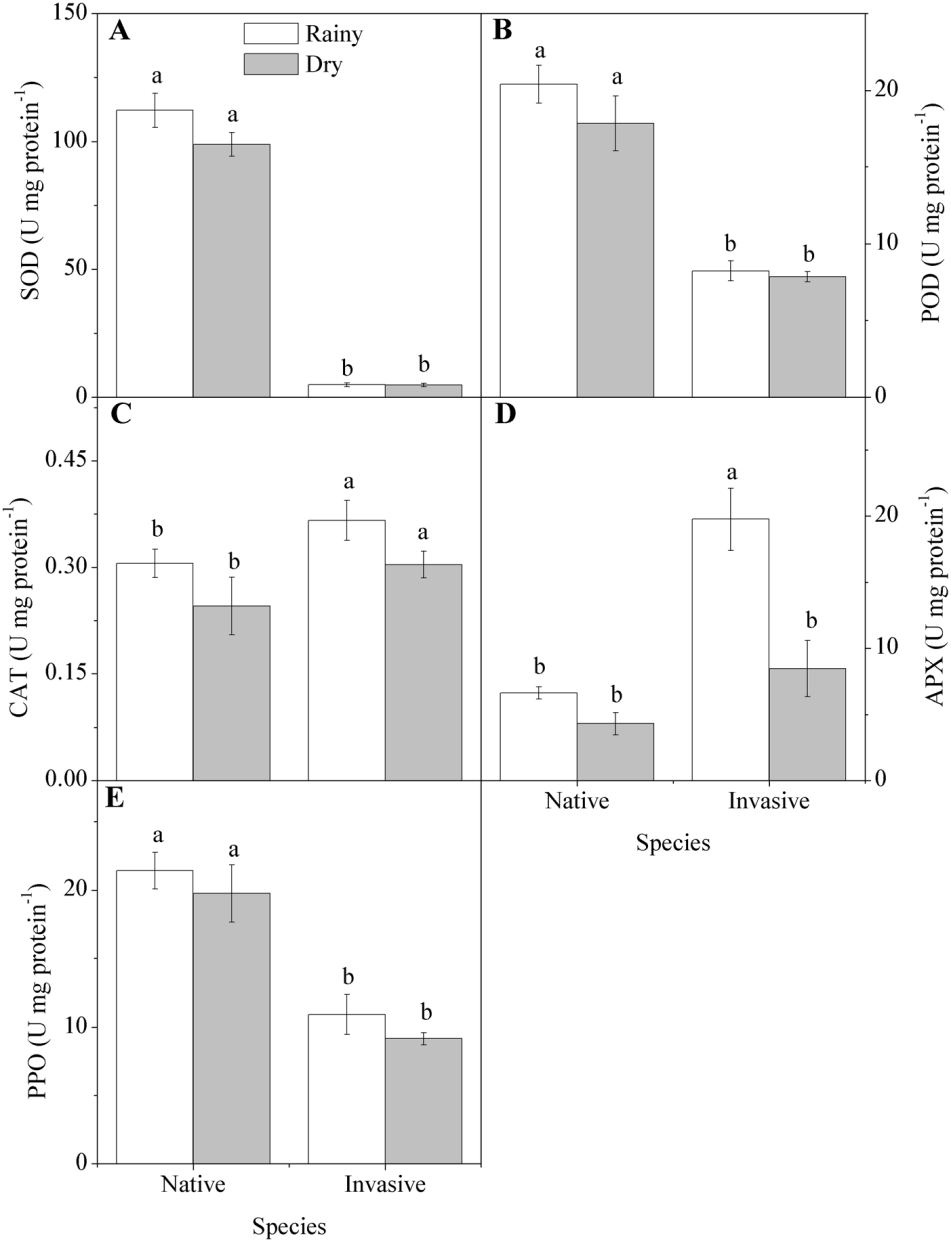
Enzyme activity in native (*Anadenanthera colubrina)* and invasive (*Prosopis juliflora*) species in a tropical dry forest in Brazil across seasons: (A) superoxide dismutase (SOD), (B) peroxidase (POD), (C) catalase (CAT), (D) ascorbate peroxidase (APX), (E) polyphenoloxidase (PPO). Values represent the average of replicates (n = 20±SE; invasive dry season n = 25±SE). Different letters denote statistical differences by Newman-Keul test with significance level of 5 percent between variables.

With respect to the activities of enzymes involved in oxygen metabolism (Fig. 5), superoxide dismutase, peroxidase, and polyphenoloxidase had higher activities in the native species but showed no significant trends with regard to season. By contrast, the invasive species had higher activities of ascorbate peroxidase and catalase, and APX tended toward lower activity in the dry season, exhibiting a significant interaction in which the drop in enzyme activity in the dry season was considerably more pronounced in the invasive than the native species.

**Figure 6:**
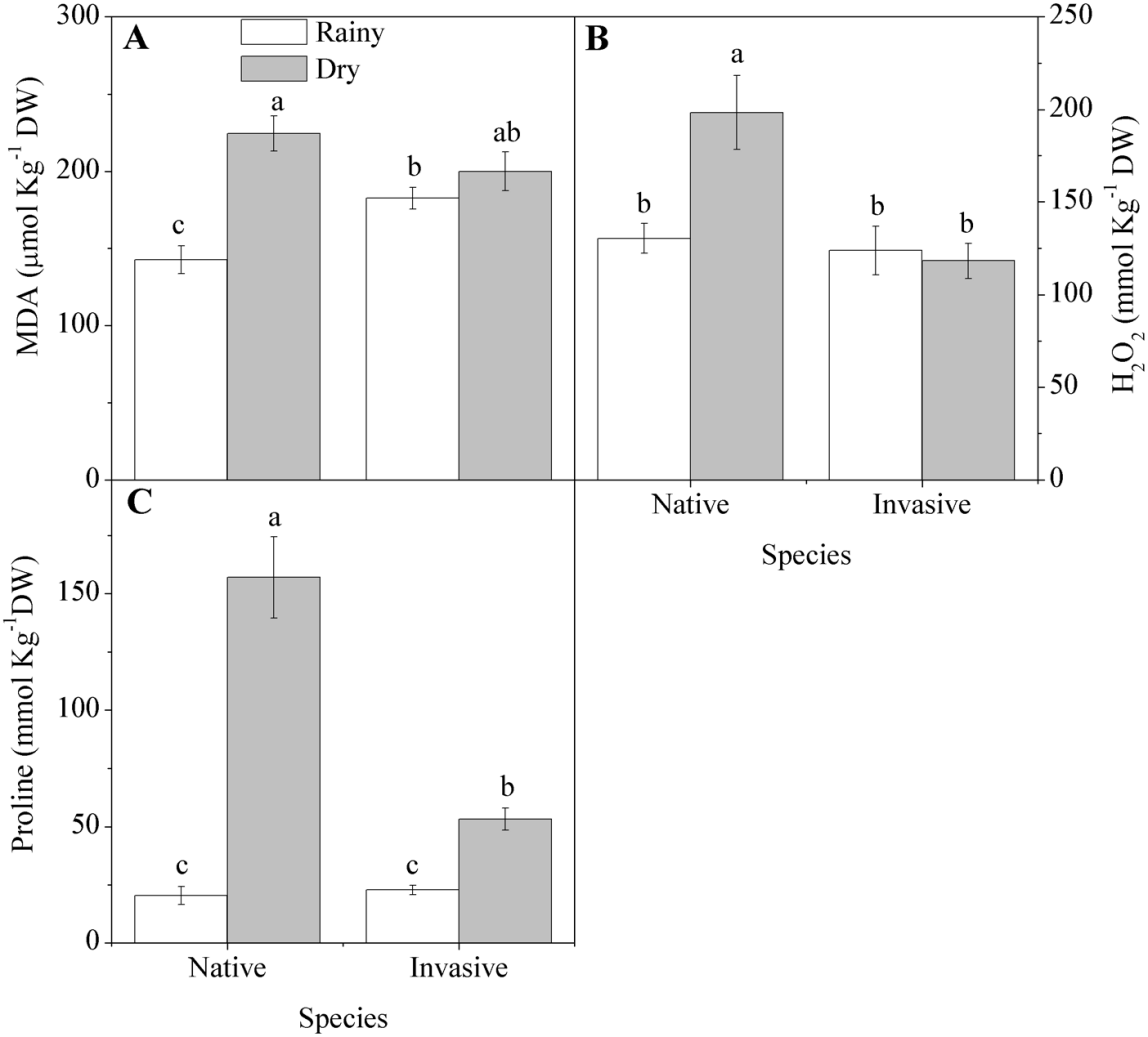
Evidence of stress in native (*Anadenanthera colubrina)* and invasive (*Prosopis juliflora*) species in a tropical dry forest in Brazil across seasons: (A) Malondialdehyde (MDA); (B) hydrogen peroxide (H_2_O_2_), (C) proline. Values represent the average of replicates (n = 20±SE; invasive dry season n = 25±SE). Different letters denote statistical differences by Newman-Keul test with significance level of 5 percent between variables.

Drought conditions promoted an increase in free radicals indicated by 58 and 52 percent higher concentrations of hydrogen peroxide and MDA in the leaves of the native species, whereas the invasive showed no changes due to season, except that the concentration of MDA concentration was 28 percent higher in the rainy season than the native (Fig. 6A and B). As expected, the proline concentration increased in both species during the dry season, but the increase was 8-fold in the native species and only 2-fold in the invader (Fig. 6C).

Foliar nitrogen was consistently higher in the invader, with values 21 percent and 9 percent higher in the rainy and dry season (Table 2). Phosphorus content did not differ between species or seasons. Foliar potassium was 125 percent higher in the invasive species in the rainy season, but species differences were not evident in the dry season.

**Table 2:** Nutrient content in leaves of native (*Anadenanthera colubrina*) and invasive (*Prosopis juliflora*) species in a tropical dry forest in Brazil across seasons.

Nutrients	Native		Invasive	
	Rainy	Dry	Rainy	Dry
N (mg g^−1^DW)	11.95±0.66^b^	13.77±0.77^b^	14.50±0.79^a^	15.14±1.19^a^
P (mg g^−1^DW)	2.51±0.34^a^	3.14±0.19^a^	3.34±0.20^a^	2.81±0.16^a^
K (mg g^−1^DW)	6.87±0.71^c^	10.20±0.58^b^	15.52±1.18^a^	11.88±0.78^b^

*Different letters on the same line denote statistical differences by Newman-Keul test with significance level of 5 percent between variables.*

Values represent the average of replicates (n = 5±SE).

## Discussion

In seeking to understand how an invader, *P. juliflora*, had replaced a seasonally dry tropical forest dominant, *A. colubrina*, we asked if *P. juliflora* exhibited a superior resource capture capacity under favorable conditions or a superior stress tolerance under unfavorable conditions. Our evidence suggests that *P. juliflora* does both.

### Biophysical Parameters

During the rainy season, the invasive species had similar rates of conductance but higher carbon assimilation than the native species (Fig. 2A and C), suggesting greater light-use efficiency on the part of the invader [35]. Several prior studies have found that the photosynthesis rates of invasive species are higher than those of natives [36]–[39]. This difference is associated with higher concentrations of foliar nitrogen found in the invasive. Increased allocation of nitrogen for photosynthesis, which can be confirmed by the higher concentration of nitrogenous products of photosynthesis as amino acids and proteins, indicate advantageous trait acquisition resources in an environment with low resource availability. These high rates of carbon assimilation coupled with high leaf N suggest a position at the “rapid-return” end of the so-called leaf economics spectrum [40].

Under favorable conditions, there was no difference in water potential and water-use efficiency between the native and the invasive species, which was not surprising given that the parameters are driven by water stress. However, under stressful conditions, while water potential reductions occurred in both species, the invader showed lower reductions due to higher water-use efficiency [2], [41]–[43]. This higher efficiency has been associated in low resource environments with greater investment in mesophyll tissue (which contains the photosynthetic machinery). Reduced allocation to structural tissue [44], indicated by the smaller amount of carbohydrates, results in lower leaf construction costs, another competitive advantage. Low construction cost is often associated with higher growth rates in plants [45], because the resources are available to produce more photosynthetic tissue, which maximizes the assimilation of carbon. Both species showed tolerance to drought by reducing water potential and increasing WUE, but the relatively higher WUE of the invasive is a strong competitive advantage in this drought-prone ecosystem. Higher efficiency of water use by invaders when compared to non-invasive congeners has been recorded in *Acer* [46] and *Rubus* [37].

### Biochemical Parameters

During stressful conditions, both the invasive and native species showed reductions in reserve compounds, but the native species increased its content of carbohydrates while the invasive decreased carbohydrates. The increase in carbohydrate by native species could play a role in scavenging reactive oxygen species [20]. This is combined with a higher concentration of carotenoids, that act to dissipate excess energy as heat [47]. Nutrient concentrations in leaves differed between the native and the invasive species, showing a greater capacity for nutrient acquisition, which may be linked to *Prosopis*’s root characteristics [48]. Microbial associations that enable better capture of the phosphorus and nitrogen for plant growth are also known among invasive trees in caatinga and other tropical dry forests [49]. Greater efficiencies in nutrient uptake have been reported for invaders in the genus *Pinus* [36], *Acer* [46] and *Rubus* [37] in addition to a diverse set of Hawaiian species [50].

The native species had higher activities of SOD, PPO, and POD, while the invasive had higher activities of APX and CAT. Investment in APX over POD, therefore, represents a strategy of protection for the organelles involved in photosynthesis, as opposed to another strategy such as preventing membrane degradation [51]–[55]. On the other hand, the invasive species performed more investment in growth and protection of the photosynthetic apparatus, while the native invested more in protecting the cell walls. Thus, under lower defense spending in the cell wall, the invasive species had higher concentrations of MDA in the rainy season than the native. The concentration of hydrogen peroxide showed an increase in the dry season only for the native, but without an increase in enzyme activity promoting a disproportionate increase in MDA concentration, this likely represented the degradation of the membranes due to lipid peroxidation. Since this peroxidation is mediated by reactive oxygen species, our results indicate that native species need greater investment in the activity of the enzymes that regulate oxygen metabolism, to keep reactive oxygen species at low levels and avoid further damage to organelles and membranes.

Neither species altered the total concentration of amino acids, but both showed changes in the type of amino acid, with an increase in both invader and native of the concentration of proline under low water availability. Proline acts as an osmotic regulator, protects against protein denaturation, stabilizes protein synthesis and sequesters free radicals [56].

The present results showed that the invasive *P. juliflora*, under favorable conditions, has advantages in resource capture, such as CO_2_ assimilation. In stressful conditions, the invasive species outperforms the native in the water-use efficiency, water potential, and elimination of free radical H_2_O_2_. For several traits the invasive maintained a consistent advantage in both seasons, exhibiting higher Chl/car and Chl a/b ratios as well as higher concentrations of leaf nitrogen, protein, and amino acid when compared with the native species. The invader had superior stress tolerance or better maintenance of physiological functions under unfavourable environmental conditions in the following ways: smaller increase in free radicals; the largest increases in the dry season of the water use efficiency; smaller reductions in water potential; higher photosynthetic rates in the rainy season with the same stomatal conductance; and higher concentrations of nitrogen and potassium. We conclude that *Prosopis juliflora* utilizes light, water and nutrients more efficiently than *Anadenanthera colubrina*, and suffers lower intensity oxidative stress in environments with reduced water availability and high light radiation such as the caatinga.

This work shows the importance of considering multiple biological attributes under realistic field conditions, rather than a few isolated traits, in understanding the performance of exotic species in new environments. We found that the success of *Prosopis juliflora,* in dynamic environments such as Brazilian caatinga, owes to a diverse set of attributes that allows the species to favor resource capture in the more resource-rich period and maintenance in stressful conditions, rather than by adopting a single strategy. More studies are needed that integrate competing hypotheses about invasion comprehensively, instead of testing them in isolation. Such a unified structure will help consolidate theory and reframe invasion as a multifaceted process.

## Supporting Information

Table S1 This table contains original data for all biochemistry measurements.
(XLSX)

Table S2 This table contains original data for all nutrient measurements.
(XLSX)

Table S3 This table contains original data for all gas exchange measurements.
(XLSX)
